# Histo-Blood Group Antigens in Oral Cancer and Potentially Malignant Disorders

**DOI:** 10.31557/APJCP.2020.21.4.1163

**Published:** 2020-04

**Authors:** Archana Pokala, Geetha Paramkusam, M L Avinash Tejasvi, Balaji Babu Bangi, Lakshmi Kavitha Nadendla, Revath Vyas Devulapalli

**Affiliations:** 1 *Department of Oral Medicine and Radiology, Kamineni Institute of Dental Sciences, Narketpally, Telangana, *; 2 *Private Practitioner,*; 3 *Dental Assistant Surgeon, Hyderabad. India. *

**Keywords:** Blood group antigens, oral cancer, potentially malignant disorders, specific red cell adherence test

## Abstract

**Background:**

Early detection of oral cancer is of critical importance because survival rates markedly improve when oral lesions are identified at an early stage. Aim of the present study is to investigate the expression of ABO (H) antigens in tissue specimens of oral cancer and potentially malignant disorders and to determine the role of ABO (H) antigens in tumour staging.

**Materials and Methods:**

A prospective study was conducted on 60 cases of oral cancer and potentially malignant diseases. Specific red cell adherence test (SRCA-test) was used for studying A, B and O (H) antigens in tissue specimens and iso-antigenicity of epithelium was graded according to degree of adherence of indicator red blood cells.

**Results:**

Among OSMF group, grade II adherence was seen in 53.3% cases, grade III in 33.3% cases, grade IV in 13.3% cases. In leukoplakia group, grade II adherence was seen in 26.7% cases, grade III adherence in 53.3% cases, grade IV adherence in 20% cases. Within the leukoplakia group, cases with dysplasia showed decreased adherence, compared with cases without dysplasia. Oral cancer group, negative adherence was seen in 13.3% cases, grade I adherence in 46.7% cases, grade II in 40% cases. In oral cancer group, antigen reactivity was less in poorly and moderately differentiated carcinoma, compared to well differentiated carcinoma.

**Conclusions:**

Antigen adherence and degree of loss of ABO (H) antigens in tissue specimens can be used for staging of the tumour.

## Introduction

Head and neck cancer is the sixth most common human cancer (Williams, 2000), representing 3% of all types of cancer. Oral cancer refers to a subgroup of head and neck malignancies that develop at the lips, tongue, salivary glands, gingiva, floor of the mouth, oropharynx, buccal surfaces and other intra-oral locations, according to the International Classification of Diseases (Shah et al., 2003). Oral cancer constitutes 48% of head and neck malignancies and 90% of these are oral squamous cell carcinoma (Jemal et al., 2009).

Early detection of cancer is of critical importance because survival rate is markedly improved when the oral cancer is identified at an early stage. Oral cancer often develops clinically as a two stage process, the first step being the appearance of a potentially malignant lesion and the second step is the development of carcinoma. Leukoplakia and OSMF are clinical changes in the oral mucosa before the development of oral cancer (Silverman et al., 1984).

The isoantigens of the ABO blood group system are not only confined to red cells, but are found on epithelial cells and in secretions. These ABH antigens are carbohydrate antigens which in epithelia are expressed in a highly regulated way that correlates with the pattern of epithelial differentiation and with cell maturation (Ravn and Dabelsteen, 2000). Profound changes in expression have been documented during epithelial cell migration in wound healing and in pathological processes such as malignant development, including oral carcinoma (Dabelsteen et al.,1998; Le Pendu et al., 2001).Thus the reduction or deletion of ABO (H) antigens in tissues of patients with pre malignant and malignant oral lesions could be possible risk markers for the susceptibility to oral cancer. 

The expression of these ABO antigens can be detected by monoclonal antibodies, so they can be used as a better objective marker of differentiation than the more commonly used subjective histological assessment. This could be a simple, cost effective method to determine the prognosis of patients and monitor probable pre-neoplastic lesions as compared to the molecular markers being used today. The present study was conducted to investigate the expression of ABO (H) antigens in tissue specimens of oral cancer, potentially malignant lesions and conditions and to determine the degree of loss of ABO (H) antigens as a marker of the tumour stage of the patient.

## Materials and Methods

A prospective study was conducted after approval by the Institutional Ethics committee on a total of 60 subjects after obtaining informed consent. Of the selected subjects 15 were with oral leukoplakia, 15 with OSMF, 15 with oral squamous cell carcinoma and 15 oral benign lesions. Blood samples were collected from all the subjects and ABO blood grouping was done by slide method. Incisional biopsy was done for all the subjects and biopsy specimens were fixed, processed, embedded in paraffin wax and sectioned to the thickness of 4-5µm with rotary microtome. All the tissues sections were subjected to (Specific Red Cell Adherence test) for the expression of ABO (H) antigens.

*Procedure of Specific Red Cell Adherence Test*

• Slides of 4-5 micron section were de-paraffinized in xylene and brought to water through graded ethanol, immersed in Tris Buffered Saline 0.05 M (pH 7.4) for 30 minutes, covered with isologous antisera according to patient’s blood group and incubated for one hour for A, B and O group in a moist chamber at room temperature.

• The slides were then dipped in Tris Buffered Saline for three times with occasional stirrings to remove the unreacted antisera.

• Few drops of 2-5% isologous indicator Red Blood Cells (RBC) suspension was added to the sections and incubated for 20 minutes for group A or B and one hour for group O.

• The slides were then inverted over a support in a Petri dish containing tris buffered saline such that the undersurface of the slide just touches the solution and kept for five minutes to settle down unreacted RBCs.

• The slides were observed under the microscope and photographed immediately.

In the present study the iso-antigenicity of the epithelium was graded according to degree of adherence of indicator RBCs as strongly positive adherence (++++/4) to negative adherence (-/0). Intermediate levels were graded as +/1 for 25% of adherence, ++/2 for 50% of adherence, and +++/3 for 75% of adherence. The obtained values were tabulated in Microsoft Office Excel 2013 and then studied, analyzed and compared using SPSS 20 software

## Results

The results obtained were statistically analyzed by one way ANOVA (Analysis of Variance) test and multiple comparisons between the groups were assessed for statistical significance using Tukey - post hoc analysis. The p- value = 0.05 was considered to be statistically significant.

**Graph 1:**
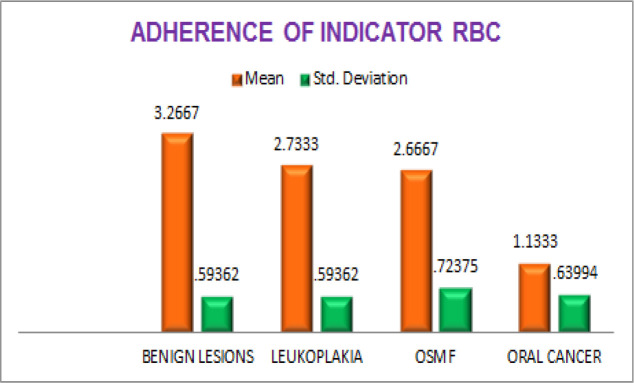
Bar Graph Showing Comparison of Adherence of Indicator RBC’s in Four Groups

The mean values for adherence of indicator RBCs in tissue sections were compared among oral benign lesions, leukoplakia, OSMF and oral cancer groups. The mean value of adherence of indicator RBCs for the oral benign lesions was 3.2667, leukoplakia was 2.7333, OSMF was 2.6667 and oral cancer was 1.1333. These mean values suggest that ABO antigens loss is minimal in benign lesions group, moderate in leukoplakia and OSMF groups and severe in oral cancer group. (Table 1 and Graph 1).

Comparison of the degree of adherence of indicator RBCs among the four groups was done by one way ANOVA test and it was found that mean value among the four groups was statistically significant (P<0.001). (Table 1).

Multiple comparisons between the 4 groups was done by Tukeys Multiple Post hoc test. ABO antigens in tissue specimens was substantially reduced in oral cancer cases when compared with OSMF, leukoplakia and oral benign lesions which was statistically significant (P<0.001). There was also significant change between oral benign lesions and oral potentially malignant disorders (P< 0.05). But there was no significant ABO antigen loss between OSMF and leukoplakia patients (P>0.05). Suggesting that there was significant loss of ABO antigens in oral cancer patients, moderate loss in OSMF and leukoplakia and mild loss in oral benign lesions (Table 2).

**Table 1:** Showing Comparison of Four Groups with Respect to Adherence of Indicator RBC’s in Tissue Sections by One Way ANOVA

	N	Mean	Standard Deviation	ANOVA *P*-value
Oral Benign Lesions	15	3.2667	0.59362	0.0001*** (among the groups)
Leukoplakia	15	2.7333	0.59362	
Osmf	15	2.6667	0.72375	
Oral Cancer	15	1.1333	0.63994	
Total	60	2.4500	1.01556	

***P<0.001

**Figure 1:**
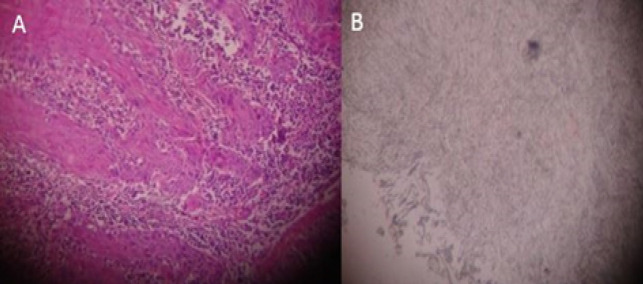
Invasive Squamous Cell Carcinoma. A, H & E staining; B, SRCA-test showing Negative antigen reactivity

**Table 2:** Showing Pair Wise Comparison of Four Groups with Respect to Adherence of Indicator RBC’s in Tissue Sections by Tukey’s Multiple Post-Hoc Procedure

Comparision Between The Groups	Mean Difference	P-Value (ANOVA- POST HOC)
BENIGN LESION vs LEUKOPLAKIA	0.533	0.026**
BENIGN LESION vs OSMF	0.600	0.013**
BENIGN LESION vs ORAL CANCER	2.133	0.0001***
LEUKOPLAKIA vs OSMF	0.067	0.7760
LEUKOPLAKIA vs ORAL CANCER	1.600	0.0001***
OSMF vs ORAL CANCER	1.533	0.0001***

*** P<0.001; **P< 0.05

**Figure 2:**
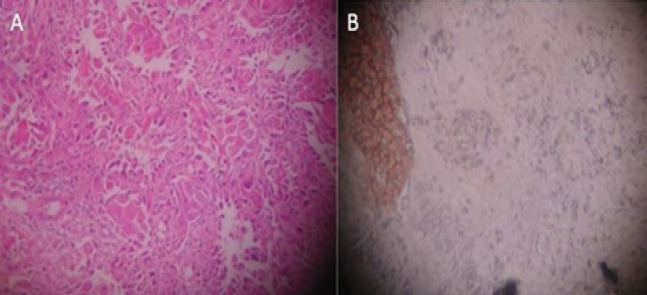
Moderately Differentiated Squamous Cell Carcinoma. A, H & E staining; B, SRCA-test showing Grade-I adherence

**Figure 3:**
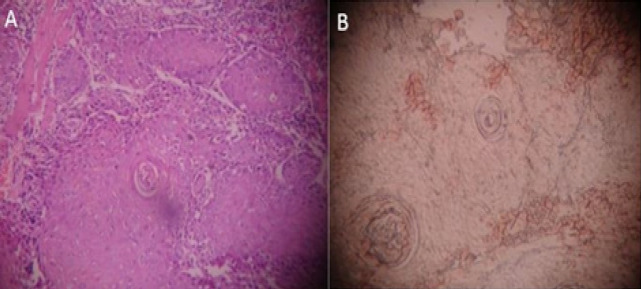
Well Differentiated Squamous Cell Carcinoma: A, H & E staining; B, SRCA-test showing Grade – II adherence

## Discussion

Although blood group antigens were initially identified as erythrocyte surface antigens and their significance was mainly ascribed to serology, it soon became evident that these antigens are widely distributed in human tissues. In fact, phylogenic studies have shown that erythrocytes are the last to acquire these antigens (Landsteiner and Miller, 1925). Soon after the discovery of the ABO system by Landsteiner in 1900, the blood group antigens were demonstrated in other cells of different organs. The antigens were found in most epithelial tissues and in secretions, and in 1928 the term “cell groups” rather than “blood groups” was suggested by Witebsky. Much later the term “histo-blood group antigens” was suggested for blood group antigens located on cells other than erythrocytes (Clausen et al., 1989).

Blood group antigens ABH are expressed most abundantly in endodermal epithelial cells (i.e. oral, esophageal, gastric, intestinal, colorectal, bronchopulmonary, urogenital), where the majority of human cancers arise. Therefore, changes in these blood group and related antigens constitute the major tumor associated changes of glycosylation and many of them lead to formation of tumor-associated carbohydrate antigens (Hakomori, 1989).

The first reports of ABO changes in tumours were described as early as 1930, and changes in expression of blood group antigens in oral carcinomas were reported by Kovarik in 1968, and later confirmed by several other authors. In normal non-keratinized oral epithelium, A/B antigens are found on spinous cells (Vedtofte, 1984). The changes seen in carcinomas are characterized by loss of A or B antigen reactivity from the carcinoma cells. Similar changes have been observed in some lesions with epithelial dysplasia. In general, tumours that retain their A or B blood group antigen specificity have a much better prognosis than tumours that do not (Dabelsteen et al., 1988; Dabelsteen et al.,1971).

The mechanisms of aberrant ABO antigen expressions in oral precancerous and cancerous lesions are not clear in all cases. In the normal oral cavity, keratinized epithelium in the palate or gingiva shows little or no expression of A or B blood-group antigen (Dabelsteen et al., 1991). Since a change from a non-keratinized to a keratinized differentiation pattern is a characteristic of many oral carcinomas and potentially malignant lesions, the lack of expression of blood- group antigens in such lesions could be due to a change in differentiation pattern of the epithelium (Dabelsteen et al., 1975). However, it has been demonstrated that half of the leukoplakia’s that developed in buccal mucosa show expression of “A” antigen, even though they histologically appear as keratinized lesions (Gao et al., 2004). Similar “A” expression was found in mechanically induced hyperkeratinized lesions of buccal mucosa. These findings indicate that loss of antigen is not invariably associated with hyperkeratinization (Dabelsteen et al., 1975).

A relative down-regulation of the glycosyltransferase that is involved in the biosynthesis of A and B antigens is seen in oral carcinomas in association with tumor development (Auclair, 1984). The events leading to loss of A transferase activity are related, in some instances, to loss of heterozygosity (LOH) involving chromosome 9q34, which is the locus for the ABO gene (Orlow et al.,1998) and in other cases, to a hypermethylation of the ABO gene promoter (Gao Set al., 2004). Hypermethylation targets only the ABO locus, but not surrounding genes, which suggests that hypermethylation is a specific tumor-related event. However, since not all situations with lack of expression of A/B antigens can be explained by LOH or hypermethylation, other regulatory factors outside the ABO promoter may be functional in transcriptional regulation of the *ABO* gene.

ABO antigens can also be present on key receptors such as EGF receptors, integrins, cadherins and CD-44, which control cell proliferation, adhesion and motility. As the expression patterns of these receptors vary in normal and cancerous cells, the role of ABO antigens in tumerogenesis may be different as well (Biondi et al., 2008).

In the present study when the tissue sections were analyzed for antigen reactivity, benign lesions always reacted positively. In OSMF group; grade II adherence was seen in 53.3% cases, grade III in 33.3% cases, grade IV in 13.3% cases. In the leukoplakia group; grade II adherence was seen in 26.7% cases, grade III adherence in 53.3% cases, grade IV adherence was seen in 20% cases. Within the leukoplakia group, cases with dysplasia showed decreased adherence when compared with the cases without dysplasia. In the oral cancer group, negative adherence was seen in 13.3% cases, grade I adherence in 46.7% cases, grade II in 40% cases (Figures 1, 2 and 3). Within the oral cancer group, antigen reactivity was less in poorly and moderately differentiated carcinoma when compared with the well differentiated carcinoma. Thus the degree of loss of ABO (H) antigens in tissue specimens can be used as a marker of the tumour stage of the patient.

The results of the present study were in accordance to several other studies in literature which were performed to investigate expression of ABO antigens in tissue sections of oral precancer and cancer (Dabelsteen et al., 1971; Gao et al., 2004; Campi et al., 2007).

In conclusion, there is significant loss of ABO antigens in tissue specimens of oral cancer cases when compared with OSMF, leukoplakia and benign lesions. Within the oral cancer group, antigen reactivity was less in poorly and moderately differentiated carcinoma when compared with the well differentiated carcinoma.
